# Mechanistic advances in factors influencing phenotypic variability in cerebral autosomal dominant arteriopathy with subcortical infarcts and leukoencephalopathy: a review

**DOI:** 10.3389/fneur.2025.1573052

**Published:** 2025-05-21

**Authors:** Ying Zhao, YaRu Lu, FengYu Wang, YaDan Wang, YaQiong Li, RuiHua Sun, JunKui Shang, Chao Jiang, Jiewen Zhang

**Affiliations:** ^1^Department of Neurology, Zhengzhou University People's Hospital, Henan Provincial People's Hospital, Zhengzhou, Henan, China; ^2^Department of Neurology, Henan University People's Hospital, Henan Provincial People's Hospital, Zhengzhou, Henan, China

**Keywords:** CADASIL, NOTCH3, granular osmiophilic material, epidermal growth factor-like repeats, phenotype

## Abstract

Cerebral autosomal dominant arteriopathy with subcortical infarcts and leukoencephalopathy (CADASIL) is a monogenic cerebral small-vessel disease caused by mutations in *NOTCH3* and is the most common hereditary cerebral small-vessel disease in adults. The clinical manifestations of CADASIL include migraines, recurrent ischemic stroke, progressive cognitive deterioration, and psychiatric symptoms. The most prevalent and earliest imaging alterations in CADASIL are white matter hyperintensities in the periventricular white matter, temporal pole, external capsule, frontoparietal white matter, and other areas on magnetic resonance imaging. Despite the substantial variations in the clinical phenotypes and disease severity in patients with CADASIL, the specific mechanisms underlying these differences remain unclear. Exploring these underlying mechanisms is crucial for enhancing our understanding of CADASIL and offering insights into its early diagnosis and treatment. This review explores the advances in research on the molecular mechanisms contributing to the variability in clinical phenotypes and disease severity among CADASIL patients with different mutations.

## 1 Introduction

Cerebral autosomal dominant arteriopathy with subcortical infarcts and leukoencephalopathy (CADASIL) is the most common inherited form of single-gene cerebral small vessel disease (CSVD) in adults. It is caused by mutations in the *NOTCH3* gene located on chromosome 19, which results in the number of cysteines within one of the 34 epidermal growth factor-like repeats (EGFr) in the extracellular structural domain of the NOTCH3 protein (1, 2). Clinical manifestations are characterized by migraine with aura, recurrent subcortical ischemic stroke, transient ischemic attacks (TIAs), psychiatric symptoms, and progressive cognitive impairments (3). Additional symptoms include Parkinsonian syndrome, atypical paraplegia, and seizures (4). On imaging, the hallmark and earliest feature of CADASIL is the presence of white matter hyperintensities (WMH) predominantly in the periventricular white matter, temporal poles, external capsule, and frontal and parietal regions on magnetic resonance imaging (MRI) (5). Lacunar infarcts, cerebral microbleeds (CMBs), and enlarged perivascular spaces are also characteristic imaging findings in CADASIL. Abnormal WMH on MRI may occur earlier than clinical symptoms. The diagnostic gold standard comprises the hallmark pathological feature—granular osmiophilic material (GOM) deposited on arterial vascular smooth muscle cells (VSMCs)—combined with *NOTCH3* gene mutations (6). Although the characteristic clinical and imaging manifestations have been delineated, accurate identification and early diagnosis of the disease remain challenging owing to the high variability of the phenotype and the incompleteness of clinical manifestations, resulting in a high rate of missed diagnosis and misdiagnosis. Moreover, the actual prevalence of *NOTCH3* mutations in the general population is higher than expected, further contributing to increased social burden (7).

Mutations in the *NOTCH3* gene have been identified as the major causative factor for CADASIL. However, phenotypic variations among patients suggest a complex interplay of genetic and non-genetic factors, such as the environment, lifestyle, and genetic modifiers. The potential interaction of these factors with the *NOTCH3* mutation on disease onset, severity, and progression has been previously documented. This review provides an overview of the potential factors that influence the severity of the clinical phenotype of CADASIL, from molecular mechanisms to genetic modifiers, offering a comprehensive overview of the phenotypic variability of CADASIL.

## 2 Molecular pathogenesis of CADASIL

The pathogenic mechanism of CADASIL is primarily attributed to missense mutations in exons 2–24 of *NOTCH3*, which lead to an abnormal number of cysteine residues, resulting in either an increase or a decrease to the odd number (8). NOTCH3, a member of the NOTCH family located on chromosome 19, consists of 33 exons that encode the highly conserved NOTCH3 transmembrane receptor. The NOTCH3 protein comprises three key domains: an intracellular domain (NOTCH3^ICD^) essential for downstream signaling, an extracellular domain (NOTCH3^ECD^) involved in ligand binding, and a single transmembrane region (9). The NOTCH3 signaling pathway plays a crucial role in regulating cell differentiation, proliferation, and apoptosis and is a critical determinant in the development of various organs (9, 10).

**Figure 1:**
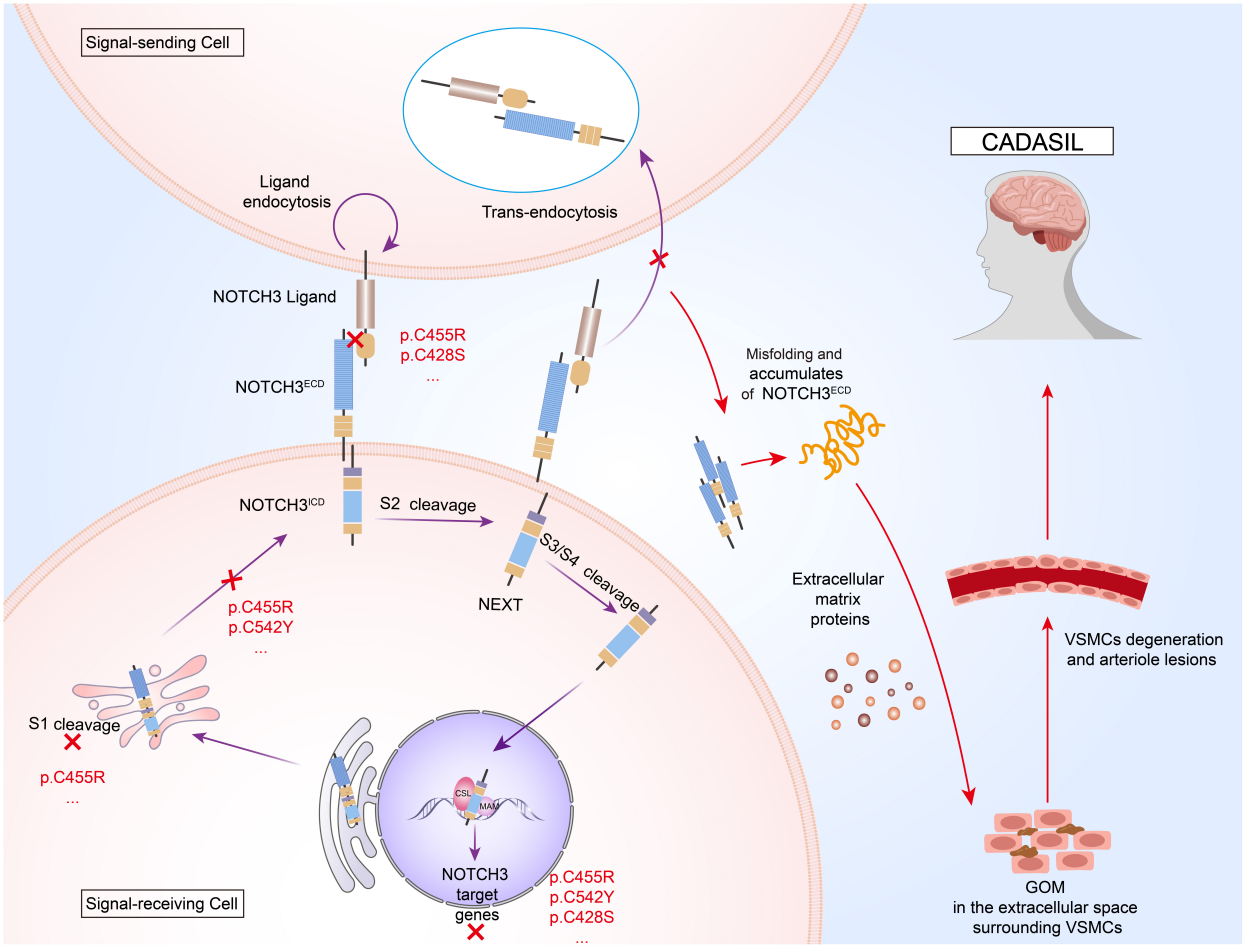
Pathogenic mechanisms of cerebral autosomal dominant arteriopathy with subcortical infarcts and leukoencephalopathy (CADASIL). NOTCH3 is a transmembrane protein comprising an extracellular domain (NOTCH3^ECD^), a transmembrane region, and an intracellular domain (NOTCH3^ICD^). After S1 processing in the Golgi apparatus, NOTCH3 protein binds to ligands on the surface of signal-sending cells to activate S2 cleavage. Ligands bind NOTCH3^ECD^ and are endocytosed into signal-sending cells. Notch extracellular truncation (NEXT) generated by S2 cleavage is further processed at the S3 and S4 sites. The released NOTCH3^ICD^ is translocated to the nucleus to bind to core binding factor 1/suppressor of hairless/longevity assurance gene 1 (CSL) and mastermind (MAM), where the three-protein complex recruits co-activators (Co-A) to activate target gene transcription. *NOTCH3* mutations result in the formation of granular osmiophilic material (GOM), extracellular aggregates that are hallmark of CADASIL, by inducing NOTCH3^ECD^ misfolding and aberrant tertiary structure conformation, resulting in aberrant dimerization of the NOTCH3^ECD^ and abnormal interactions with other proteins. Additionally, these mutations may interfere with the reduced signaling activity of ligand binding by affecting the processing of the NOTCH protein at the S1 cleavage site by Furin-like convertase in the Golgi apparatus.

The NOTCH3^ECD^ contains 34 EGFr and 3 Notch/Lin12 repeat fragments, where EGFr is essential for ligand-receptor binding (11). Each EGFr consists of ~40 amino acids, including six cysteine residues that pair to form three disulfide bonds, which are vital for maintaining and stabilizing its secondary structure (12). NOTCH3 is predominantly expressed in VSMCs and pericytes of small arteries (9). The classical NOTCH3 signaling pathway is activated by members of the Delta/Jagged transmembrane ligand family (8). After endoplasmic reticulum synthesis, NOTCH3 is processed through three sequential regulated proteolytic cleavage steps following ligand binding. This cascade culminates in the nuclear translocation of active NOTCH3^ICD^, which interacts with CSL transcription factors and mastermind (MAM) co-activators through its RBP-Jκ-associated domain to regulate downstream target gene expression (13, 14) (Figure 1).

### 2.1 GOM-associated toxicity

Cysteine-altered *NOTCH3* mutations disrupt the formation of disulfide bonds, leading to misfolding of EGFr by interfering with glycosylation of its distal structural domains (15). These structural changes alter the spatial conformation of EGFr and promote abnormal segmentation and dimerization of NOTCH3. This abnormality leads to the aggregation of NOTCH3^ECD^ in the interstitial space of the VSMCs (15, 16). Under normal conditions, NOTCH3^ECD^ enters ligand-expressing cells via endocytosis and is degraded through the transendocytic pathway. However, mutant NOTCH3^ECD^ resists effective degradation, resulting in impaired clearance (17, 18). Immunoelectron microscopy studies employing domain-specific NOTCH3 antibodies have identified NOTCH3^ECD^ as the predominant component of GOM in CADASIL patient skin biopsies (19). The NOTCH3^ECD^ cascade hypothesis posits that the elevation or accumulation of NOTCH3^ECD^ levels in vasculature serves as the central event initiating the cascade. Cellular models have demonstrated that NOTCH3^ECD^ recruits extracellular matrix proteins, including tissue inhibitors of metalloproteinase 3 (TIMP3) and vitronectin (VTN), via protein-protein interactions to form stable, extracellular complexes, which exacerbate multifactorial cytotoxic effects (20). The reduction of TIMP3 or VTN has been shown to improve the CADASIL phenotype in a transgenic mouse model (21). Furthermore, studies using a transgenic mouse model of CADASIL have suggested that the pathogenesis of the disease is likely associated with the acquisition of a toxic function of NOTCH3 (12). Structural alterations from mutations impair physiological receptor-ligand interactions while promoting pathological protein aggregation. This ultimately leads to degeneration of VSMCs and fibrosis in small arteries, contributing to disease progression (15, 22). Notably, NOTCH3^ECD^ aggregation precedes neurological symptom onset in both mouse models and human specimens, representing an early pathogenic event (23, 24). Additionally, NOTCH3 expression levels correlated with the age of onset in mice and the severity of perivascular NOTCH3^ECD^ accumulation in mouse models (25). These findings support the role of GOM-associated toxicity in the pathogenesis of CADASIL.

### 2.2 Abnormalities in the NOTCH3 signaling pathway

The most widely accepted hypothesis is that the mutant NOTCH3 protein exerts a toxic effect on VSMC membranes, contributing to the pathogenesis of CADASIL. However, the alternative hypothesis suggests that the pathogenic impact of mutations may be linked to a defect in signaling within the mutant receptor (26, 27). Some case reports have demonstrated that NOTCH3 loss-of-function mutations can induce a CADASIL-like phenotype even in the absence of GOM deposition, indicating that dysregulated signaling alone may be sufficient to cause disease (28). A previous study analyzed the signaling induced by the ligand Delta1/Jagged1 via RBP-Jκ to investigate the effects of three pathogenic *NOTCH3* mutations on receptor bioactivity. Notably, R133C and C183R mutations [outside the ligand-binding domain (LBD)] showed normal Jagged1-induced signaling in A7r5 VSMCs, while the C455R mutation (within the LBD) significantly reduced signaling activity. Ligand binding assays further revealed that the C455R mutation disrupts LBD structure, impairing Delta1/Jagged1 binding (29). Studies involving C455R *NOTCH3* gene knockout mice demonstrated that NOTCH3 signaling is both necessary and sufficient to support the coverage of arterial wall cells, and restoring NOTCH3 signaling in these wall cells effectively prevented CSVD phenotypes in mice (30). Another study investigated five naturally occurring mutations in CADASIL, of which C212S and R90C were located in the previously identified mutation hotspot region EGFr25, C428S was located in the LBD of EGFr10–11, and the remaining two mutations, C542Y and R1006C, were located in EGFr13 and 26, respectively. The study found that two mutations (C428S and C542Y) impaired receptor activity through distinct molecular mechanisms involving the RBP-Jκ transcription factor, whereas the activities of the other three mutations were unaffected (8). These findings reveal that mutations in the EGFr10–11 domain—the LBD region for delta/serrate/LAG-2 (DSL) receptors of NOTCH3—and those disrupting membrane localization significantly impair NOTCH3-mediated downstream signaling transduction, thereby compromising NOTCH3-dependent cellular functionality. However, a recent study employing a murine model demonstrated that the R169C mutation increased NOTCH3 activity. This enhancement potentially involves the disruption of endosomal trafficking induced by aberrant deposition of NOTCH3^ECD^, subsequently driving vascular structural lesions via processes including the regulation of VSMCs cytoskeleton remodeling (31). Notably, inhibition of NOTCH signaling effectively alleviates luminal stenosis, whereas conditional activation of NOTCH3 directly induces analogous structural alterations independent of hemodynamic changes. These findings suggested that excessive activation of NOTCH3 signaling constitutes the pivotal pathogenic mechanism. Based on these studies, it is hypothesized that NOTCH3 signaling functions as a signaling threshold (or Goldilocks) pathway in the context of CSVD (32). Both loss of function and excessive activation of NOTCH3 signaling are hypothesized to facilitate mural cell degeneration through downstream effects on the expression of cell survival-related genes and proteins, consequently disrupting vascular homeostasis.

### 2.3 NOTCH3 non-enzymatic cleavage and fragments

In various chronic neurodegenerative diseases, such as Alzheimer's disease, frontotemporal lobar degeneration, and amyotrophic lateral sclerosis, pathological protein fragments resulting from proteolytic cleavage, including amyloid-beta (Aβ), tau, and TAR DNA-binding protein of 43 kDa (TDP-43), are implicated in the onset and progression of these conditions (33, 34). A previous study has elucidated a novel molecular mechanism underlying vascular disease in CADASIL, diverging from the traditional NOTCH signaling pathway, which typically involves a protein hydrolysis cascade to release its transcriptionally active intracellular structural domains (35). In CADASIL patients with *NOTCH3* mutations, a specific spontaneous cleavage between EGFr1 and EGFr2 has been identified, occurring independently of the proteolytic enzymes (36). This investigation revealed site-specific fragmentation of the NOTCH3 protein through pathological analysis of the cerebral arterial vasculature in CADASIL patients and localized the fragmentation site to the NOTCH3 N-terminus at the peptide bond connecting Asp80 and Pro81. This non-enzymatic cleavage occurred within the extracellular matrix at the ultrastructural level, effectively separating EGFr1 from the remainder of the protein. The process depends on acidic pH and a reducing environment, and the enrichment of cleavage products N-terminal NOTCH3 fragments (NTF) in GOM within the media of cerebral arteries of CADASIL patients was confirmed using specific antibodies. Electron microscopy analysis revealed that the cleavage products colocalized with the basement membrane, collagen fibers, and GOM, while mutations of multiple cysteine residues of *NOTCH3* significantly accelerated the cleavage by disrupting the stability of disulfide bonds. Subsequently, another study identified a comparable non-enzymatic cleavage of the NOTCH3 protein at Asp121, located between EGFr2 and EGFr3. This cleavage generates a peptide known as NTF2, which is markedly enriched in leptomeningeal arteries and can be inhibited by phosphate, EDTA, and other anions (37). This suggests that ion concentration in the vascular microenvironment may regulate lytic activity. Recently, a third Asp-Pro site (Asp964), located between EGFr24 and EGFr25, was identified, and its cleavage product NTF3 was found to affect disulfide integrity by altering the REDOX state of the NOTCH3^ECD^ (38). Moreover, abnormal interactions with proteins such as voltage-gated potassium channel subunits may drive vascular matrix remodeling.

All three studies highlighted that the cleavage of Asp-Pro sites shared the following common characteristics: (1) dependence on a reducing environment and specific pH conditions (Asp80/Asp121 favor acidity, whereas Asp964 cleavage remains active at physiological pH); (2) regulation of NOTCH3 by its own concentration, forming a “positive feedback loop”; (3) accumulation of cleavage products (NTF, NTF2, and NTF3) in the extracellular matrix and colocalization with matrix proteins such as collagen, potentially promoting pathological deposition by altering the local protein interaction network. These data collectively support the potential pathogenic mechanism of multi-site non-enzymatic cleavage of the NOTCH3 protein in the progression of CADASIL. The non-enzymatic cleavage of NOTCH3 at sites such as Asp80, Asp121, and Asp964 may drive the progression of CADASIL through a “multi-stage amplification” mechanism: initial cleavage products (such as NTF2) promote subsequent cleavage by disrupting redox homeostasis, forming a positive feedback loop.

However, it should be emphasized that the current evidence for these non-enzymatic cleavage phenomena is mainly based on the analysis of patient vascular tissues and *in vitro* recombinant protein experiments. The role of these phenomena in the pathophysiological process still needs to be further confirmed through models such as gene-edited animals. Future research should clarify the functional heterogeneity of different cleavage fragments and their interaction networks with matrix proteins and establish a direct dose–response relationship between the levels of cleavage products and clinical severity (such as the volume of WMH) to promote their application as prognostic markers and further explore targeted therapeutic sites.

## 3 Factors associated with variations in CADASIL genotypes and clinical phenotypes

### 3.1 Genetic determinants

#### 3.1.1 Location of NOTCH3 mutations in EGFr

Many cohort studies have consistently demonstrated regional mutation-dependent variability in clinical manifestations and prognosis among CADASIL patients. A retrospective cohort study of 664 CADASIL patients in the Netherlands and Europe and a general population database found that *NOTCH3* mutations located in EGFr1–6 (HR-EGFr) were associated with early stroke onset, poor survival, and severe WMH (39). This study proposes that the *NOTCH3* mutation location is a central factor in the phenotypic heterogeneity of CADASIL. This concept was further confirmed by another large cohort study, which found that patients with mutations in EGFr1–6 had a higher risk of stroke, dementia, and daily life dependence than those with mutations in EGFr7–34. Importantly, this association is independent of age, sex, and vascular risk factors (40). The C117F and C174Y variants within EGFr1–6 exemplify this pattern, demonstrating accelerated disease progression and mortality in longitudinal studies (41). Notably, EGFr7–34 mutation carriers under 50 years of age frequently show minimal CSVD burden, often presenting with normal brain MRI findings (42). Mutations in EGFr7–34 are associated with milder clinical symptoms, potentially leading to misdiagnosis or non-diagnosis. While conventional estimates suggest prevalence rates of 2–5 per 100,000, emerging population-level data indicate actual prevalence is expected to be 100 times higher than previously predicted (39, 43–45). To systematically compare the clinical and imaging distinctions between EGFr1–6 and EGFr7–34 mutations, we have synthesized key findings in Table 1.

**Table 1:** Comparison of clinical and imaging features of mutations in EGFr1–6 and 7–34 in CADASIL.

**Study**	**EGFr1–6 vs. EGFr7–34 mutations**		
	**Sample (region)**	**Clinical features**	**Imaging features**
Cho et al. (109)	391 vs. 94 (UK)	Stroke onset (years): 55 (IQR: 13) vs. 64 (IQR: 18) Stroke onset risk: HR = 2.05 (95% CI: 1.43–2.94, *p* = 8.5 × 10^−5^) Encephalopathy risk: HR = 2.70, (95% CI: 1.15–6.37, *p* = 0.02)	There was no significant difference in WMH, lacunes, microbleeds, and brain volume.
Hack et al. (110)	97 vs. 103 (Dutch)	Stroke onset (years): 58 vs. >73 (*p* = 8.1 × 10^−4^) TIA (years): 57 vs. 72 (*p* = 0.019) Earlier stroke onset risk: HR = 2.45 (95% CI:1.39–4.31, *p* = 0.002)	nLV: OR = 4.31 (95% CI: 2.31–8.04, *p* = 4.0 × 10^−9^) nWMHv: *B* = 0.81 (95% CI: 0.60–1.02, *p* = 1.1 × 10^−12^) PSMD: *B* = 0.65 (95% CI: 0.44–0.87, *p* = 1.6 × 10^−8^)
Dupé et al. (40)	283 vs. 153 (European)	Migraine with aura or isolated auras: 45.9% vs. 30.9% (*p* = 0.002) Dementia: 12.4% vs. 4.6% (*p* = 0.009) IADL < 6: 17.5% vs. 9.1% (*p* = 0.021) Stroke risk: OR = 2.11 (95% CI: 1.33–3.33) Dementia risk: OR = 4.56 (95% CI: 1.85–11.26) IADL < 6 risk: OR = 3.55 (95% CI: 1.74–7.22)	Microbleeds (≥1): 31.2% vs. 45.7% (*p* = 0.003) Degree of atrophy (BPF of ICC): 81% vs. 80% (*p* = 0.016) Number of Lacunes (≥5): OR = 1.78 (95% CI: 1.10–2.89, *p* = 0.019) There was no significant difference in WMH.
Rutten et al. (39)	153 vs. 98 (Dutch)	Hypertension: 20.8% vs. 37.9% (*p* = 0.005) Mean age at DNA test (years): 44.3 vs. 52.5 (*p* < 0.001) Median latencies until first stroke (years): 55 vs. 67 (*p* < 0.001) Mean survival time (years): 68.5 vs 76.9 (*p* = 0.004) Stroke risk: HR = 2.63 (95% CI: 1.61–4.31, *p* < 0.001)	NR
	290 vs. 122 (European)	Smoking: 29.0% vs. 14.8% (*p* = 0.002) Hypertension: 15.5% vs. 31.1% (*p* < 0.001) Mean age at MRI scan (years): 48.8 vs. 57.3 (*p* < 0.001)	nWMHv: β = −0.144 (*p* = 0.002)

EGFr, epidermal growth factor-like repeats; IQR, interquartile range; HR, hazard ratio; WMH, white matter hyperintensities; TIA, transient ischemic attack; nLV, normalized lacune volume; nWMHv, normalized WMH volume; B(β), beta coefficient; PSMD, peak width of the skeletonized mean diffusivity; IADL, Instrumental activities of Daily Living; OR, odds ratio; NR, not reported.

Studies analyzing the *NOTCH3* variant frequency odds ratio across EGFr domains in large CADASIL cohorts and population databases have stratified patients into three prognostic categories: high-risk (HR-EGFr: 1–6, 8, 11, 26), medium-risk (MR-EGFr: 9–10, 12–15, 17, 25, 27, 32), and low-risk (LR-EGFr: 16, 18–20, 23–24, 28–31, 33) (46). Results indicated that HR-EGFr variants show strong associations with severe stroke risk, elevated normalized WMH volume, and excessive NOTCH3 protein aggregation in VSMCs. Notably, although EGFr11 mutations impair ligand-dependent signaling activity, this reduction shows no direct correlation with clinical severity, further supporting the protein aggregation hypothesis rather than a signaling pathway dysfunction. Additionally, a prospective longitudinal study demonstrated accelerated disease progression in patients with HR-EGFr *NOTCH3* mutations compared to patients with MR-EGFr (47). These findings collectively underscore NOTCH3 aggregation as a central driver of *NOTCH3*-small vessel disease (*NOTCH3*-SVD) pathogenesis, suggesting that the extent of aggregation critically influences phenotypic severity. Patients with an EGFr7–34 variant have significantly lower accumulation of NOTCH3^ECD^ in the skin and brain vessels than patients with an EGFr1–6 variant. Furthermore, the levels of NOTCH3 accumulation in the EGFr7–34 group showed a positive correlation with lacune count and WMH volume (48). This study, for the first time, reveals the potential mechanism by which *NOTCH3* variant location affects disease severity by regulating protein aggregation load. However, the limited availability of postmortem brain tissue samples in this cohort highlights the need for validation in large-scale studies. This spatial pattern suggests that compared with mutations in the C-terminal region, the unpaired cysteines in EGFr1–6 mutations at the N-terminal region of the NOTCH3^ECD^ are more likely to interact with other proteins (46). These N-terminal domains demonstrate a particular affinity for extracellular matrix components, including HTRA1, VTN, TIMP3, and latent transforming growth factor beta-binding protein 1 (LTBP1). These interactions can substantially enhance the multimerization of CADASIL-related proteins, leading to a heavier vascular aggregate load and severe disease manifestations. Additionally, a study investigating NOTCH3 fragmentation in a cohort of individuals with *NOTCH3* mutations identified a strong correlation between the mutation location and the generation of the neo-epitope (36). This suggested that pathogenic mutations near these cleavage sites may enhance the cleavage propensity of the NOTCH3 protein, resulting in a more severe disease phenotype.

A retrospective study conducted in France indicated that compared with common mutations in the EGFr2–5, mutations in the EGFr10–11 were associated with milder cognitive deficits and a tendency toward a reduction in lacunar infarct volume. However, genotype-phenotype correlation analysis revealed that patients with EGFr10–11 mutations exhibited a significantly higher prevalence of cerebral WMH in brain MRI (24). *In vivo* studies have shown that stroke in patients with a mutation located in EGFr11 (C455R) occurs at an abnormally early age compared to other CADASIL populations (49). Therefore, downregulation of NOTCH3 signaling may play a role in modifying the clinical phenotype of CADASIL. These findings suggest that signaling activity is differentially influenced by various *NOTCH3* mutations in CADASIL. Although CADASIL mutations do not primarily disrupt the major functions of the classical NOTCH signaling pathway, alterations in NOTCH signaling can influence the disease phenotype to some extent. One possible explanation for this phenomenon is that NOTCH receptor activity is tightly regulated and highly dependent on the cellular context, with certain mutations only impacting receptor activity under specific conditions (50). Additionally, the pathogenicity of the mutation may arise from impaired receptor activity independent of the typical RBP-Jκ signaling pathway (8), warranting further investigation to determine whether impairment of the signaling pathway in *NOTCH3* mutants contributes to disease severity and to elucidate the specific mechanisms linking NOTCH3 signaling to the disease phenotype.

#### 3.1.2 Atypical CADASIL variant types

Most mutations (>95%) associated with CADASIL are heterozygous missense mutations in *NOTCH3*, along with splice-site mutations, insertion mutations, frameshift mutations, nonsense mutations, and small in-frame deletions (51, 52). Patients with atypical *NOTCH3* mutations often present with clinical symptoms at a later stage of life and typically exhibit incomplete penetrance. Notably, these patients often lack characteristic WMH on MRI and show only non-specific vascular injury signs—without GOM deposits—on skin biopsy (51, 53). CADASIL is characterized by *NOTCH3* mutations involving changes in cysteine residues; however, ~5% of *NOTCH3* mutations are cysteine-sparing *NOTCH3* mutations (54).

Recent studies have gradually revealed that atypical *NOTCH3* mutations (such as cysteine-sparing *NOTCH3* missense mutations) cause phenotypic heterogeneity in CADASIL through unique molecular mechanisms. A systematic review of CADASIL cases carrying suspected cysteine-sparing *NOTCH3* missense mutations found that four mutations, namely p.R61W, p.R75P, p.D80G, and p.R213K, met the preset pathogenicity criteria. Clinical phenotype analysis showed that patients carrying such mutations presented typical CADASIL symptoms (such as stroke, migraine, and dementia). Still, the proportion of WMH in the temporal pole on imaging was significantly lower than that in patients with traditional cysteine mutations (55). A Korean cohort study found that the cysteine-sparing mutation group had less involvement of the anterior temporal lobe white matter (56), and a Chinese study further confirmed that this type of mutation had a later onset age and milder temporal lobe lesions (57). This imaging finding suggests that this type of *NOTCH3* mutation may cause differences in vascular pathological damage in specific brain regions (such as the anterior temporal lobe) by affecting the tendency of protein aggregation. Another cross-population systematic review revealed region-specific genotype-phenotype associations, with significantly increased frequencies of cognitive impairment and CMBs in Asian patients with cysteine-sparing *NOTCH3* missense mutations, which is a significant difference from classic mutation CADASIL patients (58). Additionally, the clinical and radiological phenotype characteristics of *NOTCH3* cysteine-sparing mutation patients vary in different regions, reflecting the interaction between the mutation site and the genetic background of the population. *In vitro* studies using p.R75P, p.D80G, and delta88-91 showed significantly enhanced aggregation similar to cysteine mutations, further leading to VSMC degeneration. Still, the degree of GOM deposition was milder than that of classic mutations, which may explain the milder anterior temporal lobe injury (59). Moreover, functional studies have shown that such mutations may cause vascular lesions by introducing proline to disrupt β-sheet folding or promote abnormal aggregation of the NOTCH3^ECD^ rather than relying on the traditional mechanism of cysteine residue deletion (55). In addition to NOTCH3^ECD^ aggregation, mechanisms such as receptor misfolding, spatial conformational changes induced by these mutations, and impaired NOTCH3 signaling may contribute to the pathogenesis of cysteine-sparing *NOTCH3* missense mutations. Further studies are required to elucidate the specific mechanisms involved.

### 3.2 Phenotypic modifiers

Genetic analysis revealed a strong association between the CADASIL loci and various families, demonstrating the genetic homogeneity of CADASIL (60). However, notable variations in the disease phenotypes have been observed among different families. Among Caucasians, migraines are reported as the first symptom in approximately one-third of patients (61), often accompanied by imaging findings revealing abnormalities in the temporal lobe (62). Conversely, in the mainland Chinese population, migraine with aura accompanied by abnormal white matter in the temporal pole is rare (63), whereas lacunar infarction in the brainstem is more common than in their Caucasian counterparts (63, 64). A systematic review indicated that the age at the onset of CADASIL in China ranges between 20 and 73 years. The main clinical manifestations include stroke/TIA and cognitive decline; other rare symptoms include migraine, primary cerebral hemorrhage, vertigo, sensory aphasia, alopecia, tinnitus, and deafness (65). The variability in clinical phenotypes across region-specific populations cannot be fully attributed to the genotypic differences across various regions and the founder effect. This suggests that environmental factors substantially influence disease phenotypes, highlighting the need for further research to elucidate the relationship between geographical location and phenotype.

#### 3.2.1 Environmental factors

In a study on monozygotic twins with CADASIL, individuals with the same genetic background exhibited distinct clinical phenotypes. This finding underscores the potential influence of environmental factors and lifestyle on the clinical progression of CADASIL (66). CADASIL is primarily associated with heterozygous mutations in the *NOTCH3* gene; however, homozygous mutations have also been identified. Compared with age-matched CADASIL patients with the R133C mutation, homozygous patients experienced an earlier first stroke, exhibited severe findings on most neuropsychological tests and MRI, and increased accumulation of GOM. However, one heterozygous patient in this study showed more rapid progression and severity than that of the homozygous patient (67). These findings highlight the critical role of environmental and genetic factors in influencing the CADASIL disease phenotype (68–70).

Studies have shown that smoking is independently associated with an earlier age of onset and may increase the risk of stroke (71). The mechanism may involve oxidative stress components in tobacco that aggravate vascular endothelial dysfunction. Hypertension increases the risk of cerebral infarction, intracerebral hemorrhage, lacunar infarcts, and CMBs in CADASIL patients through hemodynamic changes (72). The severity and frequency of WMH increase dramatically with age and are higher in symptomatic individuals (73). Diabetes mellitus was confirmed to be associated with early stroke onset in CADASIL patients (74). In addition, individuals with affected sisters with diabetes exhibited a more severe phenotype than those with the same mutation (70). Studies of glucose metabolism in CADASIL revealed downregulation of glucose transporters (GLUTs), specifically GLUT4 and GLUT2 in VSMCs, leading to impaired glucose uptake, a mechanism that may further contribute to blood flow restriction (75). In a large-scale, community-based controlled study conducted in the United Kingdom, homocysteine was identified as an independent risk factor in Caucasian patients with CSVD, particularly ischemic leukoaraiosis (76). Additionally, high homocysteine is associated with early migraine attacks (71). Homocysteine may cause disease by impairing vascular endothelium and atherothrombosis and interfering with the synthesis and metabolic hierarchy of neurotransmitters (77). Furthermore, the extent of atherosclerosis was related to the clinical severity of CADASIL (78). In addition, the mean age of stroke onset and the median age of death are earlier in men than in women (41, 79, 80). The prevalence and phenotype of pre-monocular symptoms differed between sexes. These findings suggest that hormonal status may play a role in modulating susceptibility in CADASIL patients. These findings collectively highlight that CADASIL manifestations arise not only from genetic determinism but also from dynamic interactions among *NOTCH3* mutations, vascular risk exposures, and metabolic dysregulation. As summarized in Table 2, prospective studies across diverse populations consistently report high prevalence rates of modifiable vascular risk factors in CADASIL cohorts, further validating their role as critical accelerators of disease severity.

**Table 2:** Prevalence of vascular risk factors in patients with CADASIL as reported in prospective studies.

**Study**	**Patients (*N*)**	**Region**	**Age (years)**		**Female**	**Hypertension**	**Diabetes**	**Active or past smoking**	**Hypercholesterolemia**	**Hyperhomocysteinemia**	**Atherosclerosis**
Singhal et al. (71)	127	British	48 (21–82)		61%	20%	4%	52%	45%	9%	NR
Mawet et al. (78)	144	Paris	52.68 ± 11.9		50%	16.7%	2.1%	48.3%	45.5%	NR	33.3%
Gunda et al. (80)	313	Paris	51 ± 11.4		55%	21.06%	2.22%	22.01%	41.82%	NR	NR
Bianchi et al. (111)	229	Italy	57.8 ± 14.7		48.91%	35.6% (67/188)	12.7% (24/188)	15.6% (25/160)	34.6% (65/188)	15.80% (25/158)	NR
Chabriat et al. (112)	290	Paris, Munich	50.6 ± 11.4		55.2%	19%	2.1%	20.3%	38%	NR	NR
Hack et al. (110)	200	Dutch	EGFr1–6: 48.9 ± 12.3	EGFr7–23: 55.6 ± 11.3	53%	25.5%	6.5%	50.5%	37.5%	NR	NR
Dupé et al. (40)	446	France	24–83		55.83%	28%	6.7%	61%	49%	NR	NR
Hack et al. (43)	179	Japan	54.7 ± 10.5		50.8%	23.0%	5.7%	40.9%	30.6%	NR	NR
Chen et al. (97)	216	China	49 ± 9		55.09%	22.5%			NR	NR	NR
Ospina et al. (74)	90	USA	35.5(IQR:21)		54.4%	16.6%	5.6%	33.3%	15.6%	NR	NR

NR, not reported; EGFr, epidermal growth factor-like repeats; IQR, interquartile range.

#### 3.2.2 Epigenetic regulation

In addition to *NOTCH3* mutations and environmental factors, epigenetic modifiers play a significant role in the clinical symptoms, imaging findings, and pathological changes observed in CADASIL patients (68). These genetic modifiers participate in autoregulation and neuronal responses to ischemia, as well as in repair processes and other functions at various levels of vascular endothelial and smooth muscle cells, thereby exerting a regulatory influence on CADASIL (81). Epigenetic modifications, including DNA methylation and RNA or histone modification, regulate gene expression through mechanisms such as methylation, phosphorylation, and ubiquitination. Consequently, these modifications are crucial for the growth, differentiation, and proliferation of various cell types (82). Epigenetic modifications play a critical role in the initiation and progression of neurodegenerative disorders such as Alzheimer's disease and Parkinson's disease (83–86). In a naturally occurring exon 9 skipping CADASIL family, individuals with exon 9 skipping exhibited a milder phenotype of small-vessel disease than that observed in most CADASIL patients, with significantly reduced NOTCH3^ECD^ protein aggregation in the skin. Inducing exon 9 skipping in a cellular model revealed that cysteine-corrected *NOTCH3* exon skipping reduced NOTCH3 aggregation and resulted in an attenuated phenotype (87). Arginine methylation is a common post-translational modification that plays a crucial role in various biological processes, including signal transduction, metabolism, and development within the human body. Elevated levels of asymmetric dimethylarginine following the methylation of arginine residues are significantly associated with an increased risk of adverse vascular events in CADASIL patients (88). Additionally, D-loop methylation is markedly reduced in the mitochondrial DNA (mtDNA) of CADASIL patients, further contributing to increase in mtDNA and mitochondrial dysfunction (89).

#### 3.2.3 Polygenic interactions

Emerging evidence underscores the role of polygenic factors in modulating CADASIL severity beyond *NOTCH3* mutations. The coexistence of the *NOTCH3* pathogenic variant (p.G420C) and the *SQSTM1* pathogenic variant was identified in two siblings with CADASIL. The presence of the *SQSTM1* gene as a genetic modifier may play a role in modulating NOTCH3 signaling, ultimately resulting in pronounced deterioration of the clinical manifestations associated with CADASIL in these siblings (90). Furthermore, the *apolipoprotein E (APOE)* ε*2* allele is associated with a higher WMH volume, and individuals carrying the *APOE* ε*2* allele may have more severe cognitive impairment (91, 92). A genome-wide association study reported that the polygenic score of CADASIL was associated with WMH volume, and multiple common genetic variants with small effect sizes affected WMH burden (93). Additionally, specific CADASIL alleles may increase the likelihood of corpus callosum lesions or the rate of disease progression (41, 94). It is noteworthy that CADASIL patient populations exhibit region- and ethnicity-specific mutation hotspots. For instance, in most European patients, such as British and German patients, *NOTCH3* mutations are predominantly located in exons 2–6, particularly exon 4 (95, 96). In contrast, mutations in exons 11 and 4 are more prevalent in Asian regions such as China and South Korea (63, 72, 97). Clinically, Asian patients are more prone to TIA/ischemic stroke and cognitive impairment, while Caucasians have a higher incidence of migraine and mental disorders (98). Similar to typical CADASIL, the clinical and radiological phenotypic features of *NOTCH3* cysteine retention mutation patients vary in different regions. Asian patients with atypical mutations often present a “radiological phenotype preceding clinical symptoms” pattern, with more significant imaging brain phenotypes, especially lacunes and CMBs, while Caucasians mainly exhibit typical clinical phenotypes (58). This highlights the interplay between founder effects and localized environmental pressures in shaping regional genetic landscapes. These findings collectively emphasize that CADASIL heterogeneity arises from a complex interplay of major *NOTCH3* mutations, modifier genes, and population-specific genetic drift. Future studies should integrate multi-dimensional analytical approaches, including gene-gene interactions, epigenetic regulation, and environmental influences, to elucidate the precise mechanisms underlying genotype-phenotype associations.

## 4 Modifications in clinical diagnostic criteria for CADASIL

Due to the absence of reliable biomarkers, CADASIL diagnosis primarily relies on clinician-developed criteria, necessitating a thorough understanding of its clinical heterogeneity. Given CADASIL's phenotypic variability, the diagnostic criteria initially proposed by Davous have been iteratively refined to capture its diverse manifestations (3). The screening of patients for CADASIL typically involves a combination of characteristic clinical manifestations, imaging findings, and family history. A CADASIL scale scoring system was proposed for the genetic testing population, including eight categories: migraine (with or without aura), stroke/TIA, early-onset symptoms (≤ 50 years), psychiatric disorders, cognitive decline/dementia, leukoencephalopathy (temporal pole or external capsule involvement), subcortical infarction, and family history. This scale is mainly used to identify patients with a high probability of being affected by genetic testing, with a sensitivity of 96.7% and a specificity of 74.2% (99). Over time, the original CADASIL scale has been adapted by researchers to better address the specific needs of different populations and clinical settings. The CADASIL Scale-J, adapted for Japanese cohorts, comprises eight items: hypertension, diabetes mellitus, age at onset (≤ 50 years), pseudobulbar palsy, stroke/TIA, family history, subcortical infarction, and temporal pole lesions. The sensitivity and specificity of the CADASIL Scale-J were 78.9% and 85.7%, respectively (79). These modifications aim to enhance the scale's applicability and diagnostic accuracy within specific demographic groups. Currently, typical neuroimaging hallmarks of CADASIL include T2-WMHs in the outer temporal capsule and anterior pole, multiple lacunar infarcts, and extensive demyelination (73, 100). Nevertheless, some atypical imaging findings of CADASIL, such as corpus callosum lesions (101) and CMBs (102), have been detected in an increasing number of patients, and their influence on disease severity and prognosis should not be overlooked (103, 104). Additionally, a study conducted in Slovakia identified 23 pathogenic variants in 35 unrelated families. Among them, the mutation causing the genetic defect was found in 10.2% of patients with clinically suspected CADASIL who were eventually diagnosed with CADASIL (105). Therefore, *NOTCH3* gene screening criteria should not be restricted to patients with high risk for CADASIL, especially those without typical imaging findings but presenting with early-onset stroke, a family history of stroke or dementia, and no hypertension. Such patients may benefit from a more comprehensive analysis of their clinical phenotype. In cases where diagnosis remains challenging, a skin biopsy should be performed to assist in the diagnosis.

Furthermore, the assessment of disease severity frequently depends on standardized clinical metrics (e.g., neuroimaging scores) and the assessor's personal judgment. To better analyze and compare the relevant data, a relatively consistent criterion is necessary to measure the severity in all patients. Some researchers have proposed a CADASIL severity grading system that encompasses five levels: grade 0 (absence of symptoms), grade 1 (migraine alone), grade 2 (stroke, TIA, or mild cognitive impairment), grade 3 (requiring walking assistance or dementia), and grade 4 (bedridden or advanced stage) for patients with known pathogenic *NOTCH3* mutations, characteristic ischemic lesions on brain MRI, or characteristic intravascular deposits on skin biopsy (106). Some studies have validated the scoring system regionally, with relatively promising outcomes (107). More recently, a *NOTCH3*-SVD staging system was developed for CADASIL, the classic cysteine-altered *NOTCH3* variant, to better assess disease severity and monitor disease progression. The *NOTCH3*-SVD staging system further refines this into nine substages (stages 0 to 4B), offering a standardized framework for monitoring progression (108). However, the reliability and applicability of the scoring system require further validation in more diverse patient populations.

## 5 Conclusion

CADASIL is the most common genetic cause of dementia in adults, characterized by significant phenotypic variability among patients. Over recent decades, studies utilizing animal models, cellular systems, and patient cohorts have provided valuable insights into the pathogenic mechanisms underlying CADASIL. This review primarily focused on elucidating the relationship between CADASIL and its clinical phenotypes and identified at least five factors associated with the severity of clinical manifestations in CADASIL: mutation location, variant types, environmental factors, epigenetic regulation, and polygenic interactions. Additionally, the review emphasizes the importance of refining clinical diagnostic criteria for CADASIL to improve the assessment of disease severity. Despite these advances, several potential mechanisms remain unexplored. Future studies should prioritize combining gene editing methods with multi-omics analysis to dissect the downstream molecular mechanisms caused by *NOTCH3* mutations. In addition, related studies of biomarkers are also of great significance for predicting patient phenotype and prognosis. Continuous research on the genotype-phenotype correlations in CADASIL is crucial for unraveling its heterogeneity, identifying therapeutic targets, enabling early diagnosis, and mitigating disease progression. These efforts are expected to support the development of precision medicine approaches tailored to CADASIL.

## Data Availability

This review does not involve original data collection. However, the data used to support the findings of this review are publicly available from the following sources: Gene Expression Omnibus (GEO): all relevant gene expression data discussed in this review can be accessed from the GEO database (https://www.ncbi.nlm.nih.gov/geo/). PubMed: relevant articles and publications used in this review are available through PubMed (https://pubmed.ncbi.nlm.nih.gov/). Protein Data Bank (PDB): structural data referenced in this review can be accessed through the PDB (https://www.rcsb.org/). Data sharing and accessibility were considered in the preparation of this review, and all cited data are publicly available for further research and analysis.
